# Supplementary material for: Reduced mortality in COVID-19 patients treated with colchicine: Results from a retrospective, observational study
Source: PLoS One. 2021 Mar 24;16(3):e0248276. doi: 10.1371/journal.pone.0248276 (PMC7990208; doi:10.1371/journal.pone.0248276)
Supplement: S1 Table — (DOCX) [file pone.0248276.s001.docx]

**S1 Table. Demographic and clinical characteristics of the study population after propensity-score weighting.**

|  | Control  (N = 66) | Colchicine  (N= 66) | P value# |
| --- | --- | --- | --- |
| *Comorbidities* |  |  |  |
| Diabetes | 8 (12.9) | 7 (10.9) | 0.66 |
| Cancer | 4 (6.6) | 3 (5.3) | 0.72 |
| Hypertension | 33 (50.5) | 31 (47.5) | 0.68 |
| CKD | 11 (16.3) | 12 (18.7) | 0.78 |
| *Disease severity at diagnosis* | | | |
| Non-hospitalized | 15 (23.4) | 15 (22.6) | 0.78 |
| Hosp. w/o O_2_ | 14 (21.7) | 10 (15.4) |  |
| Hosp. with O_2_ | 23 (34.4) | 25 (38.0) |  |
| Hosp. with NIV | 9 (13.0) | 12 (18.8) |  |
| *Clinical characteristics at diagnosis* | | | |
| Fever | 60 (91.4) | 61 (92.9) | 0.67 |
| Dyspnea | 27 (40.8) | 32 (49.2) | 0.46 |
| Cough | 42 (63.5) | 39 (59.0) | 0.52 |
| Arthro-myalgias | 10 (14.8) | 9 (13.1) | 0.79 |
| Diarrhea | 3 (4.8) | 5 (7.3) | 0.48 |
| Lab values at hospital admission (Inpatients) | | | |
| COVID-19 Treatment | | | |
| Use of antibiotics | 42 (63.0) | 47 (71.4) | 0.47 |
| Antiviral treatment | 36 (55.1) | 36 (54.2) | 0.82 |
| Hydroxychloroquine | 41 (62.7) | 42 (63.7) | 0.95 |
| i.v. steroids | 10 (14.5) | 15 (22.1) | 0.39 |
| Tocilizumab | 3 (4.9) | 6 (8.6) | 0.40 |

Data are expressed as number (%)

Counts in the weighted cohort may not sum to expected totals owing to rounding. Percentages may not total 100 because of rounding, and disagreements between numbers and percentages in the weighted cohort are the result of rounding of non-integer number values.

# The P values were calculated by inverse probability of treatment–weighted logistic-regression models.

CKD, chronic kidney disease; NIV, non-invasive mechanical ventilation; Hosp., hospitalized; O_2_, supplemental oxygen; i.v., intravenous
