# Supplementary material for: Reduced mortality in COVID-19 patients treated with colchicine: Results from a retrospective, observational study
Source: PLoS One. 2021 Mar 24;16(3):e0248276. doi: 10.1371/journal.pone.0248276 (PMC7990208; doi:10.1371/journal.pone.0248276)
Supplement: Attachment — Submitted filename: R1_Rebuttal.docx [file pone.0248276.s004.docx]

ID: **PONE-D-20-29570**

Title: **Reduced mortality in COVID-19 patients treated with colchicine: results from a retrospective, observational study**

**Rebuttal letter**

Journal requirements:

1. Please ensure that your manuscript meets PLOS ONE's style requirements, including those for file naming.

**We modified the manuscript to meet PLOS ONE’s style requirements.**

2.	Please note that all PLOS journals ask authors to adhere to our policies for sharing of data and materials: [https://journals.plos.org/plosone/s/data-availability](https://emea01.safelinks.protection.outlook.com/?url=https%3A%2F%2Furldefense.proofpoint.com%2Fv2%2Furl%3Fu%3Dhttps-3A__journals.plos.org_plosone_s_data-2Davailability%26d%3DDwQGaQ%26c%3DshNJtf5dKgNcPZ6Yh64b-A%26r%3DueJex95s5vG9PYhWDvL-ZvABIbymURJgIR0m4c99QaI%26m%3DKjfBy9bVlV9g3dWHea_o5T7qYbsXsKOyvx6sfIcpZlI%26s%3D-UnDE6tSeyBva89qlpe7qafaf8JQw_xhns5gvPGyk8g%26e%3D&data=04%7C01%7C%7C03d9dc082e44476c1b9208d8b85dc36c%7C84df9e7fe9f640afb435aaaaaaaaaaaa%7C1%7C0%7C637462060149427920%7CUnknown%7CTWFpbGZsb3d8eyJWIjoiMC4wLjAwMDAiLCJQIjoiV2luMzIiLCJBTiI6Ik1haWwiLCJXVCI6Mn0%3D%7C1000&sdata=J%2BKKV%2BTD4bnurbIErREOIQMTWxv4uX%2FvZDInYHLG4to%3D&reserved=0). According to PLOS ONE’s Data Availability policy, we require that the minimal dataset underlying results reported in the submission must be made immediately and freely available at the time of publication. As such, please remove any instances of 'unpublished data' or 'data not shown' in your manuscript and replace these with either the relevant data (in the form of additional figures, tables or descriptive text, as appropriate), a citation to where the data can be found, or remove altogether any statements supported by data not presented in the manuscript.

**We have reported the data in the amended version of the results.**

Furthermore, this is a retrospective study. As such, we do not feel that any conclusions on the intervention effects can be supported; as such, we ask that you revise the text to avoid unsupported statements.

**We modified the abstract conclusions accordingly. We verified that the rest of the manuscript does not include any statement implying causation.**

3.	We note that you have included the phrase “data not shown” in your manuscript. Unfortunately, this does not meet our data sharing requirements. PLOS does not permit references to inaccessible data. We require that authors provide all relevant data within the paper, Supporting Information files, or in an acceptable, public repository. Please add a citation to support this phrase or upload the data that corresponds with these findings to a stable repository (such as Figshare or Dryad) and provide and URLs, DOIs, or accession numbers that may be used to access these data. Or, if the data are not a core part of the research being presented in your study, we ask that you remove the phrase that refers to these data.

**We have reported the relevant data in the amended results section of the manuscript.**

4.	Please include your tables as part of your main manuscript and remove the individual files. Please note that supplementary tables (should remain/ be uploaded) as separate "supporting information" files

**Tables are now embedded in the manuscript.**

5. Please include captions for your Supporting Information files at the end of your manuscript, and update any in-text citations to match accordingly. Please see our Supporting Information guidelines for more information: [http://journals.plos.org/plosone/s/supporting-information](about:blank).

**We listed the supporting Information at the end of the manuscript.**

Editor Comments

1. A sensitivity analysis excluding patients using other ant-inflammatory drugs should be performed.

**During the study period no anti-inflammatory agent besides steroids, colchicine and tocilizumab, was used as for the standard of care at that time. We specified this issue in the amended version. On the other hand, sensitivity analysis for steroids and tocilizumab is reported in Fig 2.**

2. Please discuss more in details the findings regarding the effect of colchicine in patients not requiring O2 therapy.

**We added a comment on this in the amended version of the discussion section.**

3. The introduction is too wordy. Please consider shortening it.

**We shortened the introduction according to the Editor’s advice.**

4. please provide the mean dose of colchicine and describe how many patients received 0.5 mg vs 1 mg. There was any difference in outcomes?

**In the amended version of the results section we specified that the colchicine starting dose of 0.5mg was reserved to patients with drug-to-drug-interactions and/or chronic kidney failure in whom 0.5mg daily correspond to 1mg daily or more. With the limitations of different baseline comorbidities, we did not detect any sign that 0.5mg was associated with increased mortality (1 death out of the eleven patients, i.e. 9%)**

**We thank the Editor for the helpful comments that helped us improving the manuscript.**

Reviewers' comments:

1. Abstract: Please add the number of patients (both groups) in method.

**We included the number of patients of the unweighted cohort in the revised abstract.**

2. Please explain more about the severity of diseases in both groups? whether the severity of the patients on colchicine were higher than control group?

**We added a comment on the difference in the unweighted cohort in the amended version of the results.**

3. Please explain more about the history of other drugs or supplements consumption in both groups. Did patients take any drug with colchicine Simultaneously which may affect the result?

**During the study period, no anti-inflammatory agent besides steroids, colchicine, and tocilizumab, were used as for the standard-of-care at that time. We specified this issue in the amended version. Sensitivity analysis for steroids and tocilizumab is reported in Fig 2.**

4. There is not any information about the weight and Body Mass Index of patients which can affect the results (since obesity is a risk factor for covid-19 patients). It will be better to add the Antropometric measurements if they are available?

**Unfortunately, height was not available in a substantial proportion of patients. Therefore, in these patients, BMI was not available. However, we added available BMI data in the amended version of Table 1. There was no significant difference between groups.**

5. Discussion: please explain more that how the colchicine may reduce the death in COVID-19 patients according to documents?

**Revised version of the introduction and discussion include statements on the potential mechanisms responsible for the beneficial effects of colchicine on COVID-19 associated mortality.**

**We thank the Reviewer for the constructive feedback that helped improving the quality of the manuscript.**
