# Supplementary material for: Reduced mortality in COVID-19 patients treated with colchicine: Results from a retrospective, observational study
Source: PLoS One. 2021 Mar 24;16(3):e0248276. doi: 10.1371/journal.pone.0248276 (PMC7990208; doi:10.1371/journal.pone.0248276)
Supplement: Attachment — Submitted filename: Rebuttal_PC_UM.docx [file pone.0248276.s005.docx]

ID: **PONE-D-20-29570**

Title: **Reduced mortality in COVID-19 patients treated with colchicine: results from a retrospective, observational study**

**Rebuttal letter**

**Reviewer #2:**

Manenti et al. in their paper report an interesting retrospective analysis exploring the prognostic effect of an antinflammatory drug widely used in other scenarios: colchicine. The authors found that after propensity score matching the treatment with colchicine was associated with a reduced incidence of death and a significant improvement. The paper is of interest, considering also the recent relevance gathered by colchicine in COVID-19. Moreover, the paper is methodically well conducted and well written.

**Response: We thank the Reviewer for the positive comments.**

The needing to discontinue colchicne in 7% of patients introduces a bias, due to a shorter duration of the treatment. Therefore, if it is not possible to replace these patients with other similarly matched patients, this limitation should be acknowledged.

**Response: We acknowledge this issue in the study limitations (line 338). We also explained that “this study drawback should have biased our findings toward the null.”**

A sensitivity analysis to make sure that a selection bias was avoided would increase the quality of the paper.

**Response: The reviewer raised an important issues that we addressed in Figure 2 when we report the results after excluding each category based on severity of disease, comorbidity, concomitant treatment.**

The match is based only on gender and age. However, in COVID infection many other factors increase the risk of adverse outcome (i.e. comorbidities). The population should be matched also for comorbidities (at least the number of comorbidities) and the COVID treatment. To overcome this limitation, I would suggest to adjust the HR of the colchicine treatment for the variables with a statistically significant difference in the two groups (i.e. disease severity, COVID treatment and dyspnoea. Otherwise this limitation should be stated.

**The reviewer raised a relevant issue that we faced (similarly to previous retrospective published study such as N Engl J Med 2020; 382:2411-2418) using the state-of-the-art methodology to control for confounding in treatment comparison in observational studies, namely inverse probability of treatment weighting based on propensity score to construct a weighted cohort of patients (see Statistical methods Lines165-187). The analysis adjusted for imbalances between groups in baseline severity, such as chronic kidney diabetes, diabetes, cancer, hypertension, and concomitant drugs. Matching for age and sex was exclusively used for sampling controls. The statistician provided the code for the main analyses as supplementary material (below).**

```
*-------------------------------------------------------------------------------
*Start analyses with inverse probability of treatment propensity-score weighting
*-------------------------------------------------------------------------------
*----------------------------------------------------+

* Start Calculating IPTW andChecking IPTW Assumptions
*----------------------------------------------------+

use COLCHICINE_DATASET, clear

#delimit ;
global ps_ivar  "c.age i.SEX
	i.grp_severity1 i.grp_severity2 i.grp_severity3 i.grp_severity4
	i.hist_ckd  i.hist_diabetes i.hist_cancer i.hist_hypertension
	i.hist_dyspnea i.hist_cough i.hist_arhtrmyalg i.hist_diarrhea
	i.hist_antibiotics_done i.antiviral i.hydroxyxhloroquine
	i.steroids i.toci_yes";
#delimit cr

#delimit ;
global ps_nivar  "age SEX
	grp_severity1 grp_severity2 grp_severity3 grp_severity4
	hist_ckd  hist_diabetes hist_cancer hist_hypertension
	hist_dyspnea hist_cough hist_arhtrmyalg hist_diarrhea
	hist_antibiotics_done antiviral hydroxyxhloroquine
	steroids toci_yes";
#delimit cr

logit colch_tp $ps_ivar

* calculate stabilized inverse probability of treatment weights
* (Austin Stat Med 2016; 35: 5642)

predict pr1, pr
gen pr0 = 1 - pr1
qui summ pr1, meanonly
local mpr1 = r(mean)
qui summ pr0, meanonly
local mpr0 = r(mean)
gen stab_ipw = .
replace stab_ipw = `mpr1' * 1/pr1 if colch_tp ==1
replace stab_ipw = `mpr0' * 1/pr0 if colch_tp ==0
drop if missing(stab_ipw)

// check common support
summ stab_ipw if colch_tp == 0
tw kdensity stab_ipw if colch_tp == 0, lcolor(black) lpattern(solid) || ///
   kdensity stab_ipw if colch_tp == 1, lcolor(maroon) lpattern(dash) || ///
   , ///
   ytitle("Density") ylabel(, angle(horizontal) format(%3.1f)) ///
   xlabel(, forma(%3.1f)) ///
   xtitle("Stabilized inverse probability of treatment weights") ///
   legend(pos(2) ring(0) rows(2) order(1 "Control" 2 "Colchicine")) ///
   scheme(s1mono)


/// Start computation of Standardized differences
teffects ipw  (death) (colch_tp $ps_ivar, logit)
tebalance summarize


foreach var of varlist ///
	$ps_nivar  {
		di _newline(3) _col(8) in gr "--------------------> Standardized difference of var: `var'"
		qui summ `var' [aw = stab_ipw] if colch_tp == 0
		local m0 = r(mean)
		local v0 = r(Var)
		qui summ `var' [aw = stab_ipw] if colch_tp == 1
		local m1 = r(mean)
		local v1 = r(Var)
		local std = (`m0' - `m1') / sqrt((`v0' + `v1')/2)
		di _col(8) in ye %3.1f `std' * 100 " %"
		 }

/// End computation of standardized differences

*-----------------------------+
* End Check IPTW Assumptions
*-----------------------------+

*---------------------------------------------------------------+
* Start Survival Analysis (21-day mortality)
*---------------------------------------------------------------+

stset FOLLOW_DATE [pw = stab_ipw], id(id) origin(admission_date) fail(death == 1) ///
 exit(time admission_date + 21)
stcox colch_tp , cluster(id)
qui test _b[colch_tp] = 0
local pval  = r(p)
local spval = string(`pval', "%4.3f")

/// make the plot

#delimit ;
global kmstuff  "risktable(, title("N at risk", size(*.7)))
	  risktable(, color("black") group(#1) size(*.7))
	  risktable(, color("maroon")  group(#2) size(*.7))
	  risktable(, rowtitle("Colchicine: No ") group(#1) size(*.7))
	  risktable(, rowtitle("Colchicine: Yes") group(#2) size(*.7))
      plot1opts(lcolor("black") lwidth(*1.0) lpattern(solid))
	  plot2opts(lcolor("maroon")  lwidth(*1.0) lpattern(dash))
	  legend(cols(1) position(11) rows(3) ring(0) size(*.7)
	  lstyle(none)
	  order(1 "Colchicine: No" 2
	  "Colchicine: Yes"))
	  ysc(range(.75 1))
	  ylab(.25 "25" .5 "50" .75 "75" 1 "100",
	  angle(horizontal) labsize(*.7) grid )
	  xsc(titlegap(5)) xlab(0(1)21, format(%3.0f) labsize(*.7))";
#delimit cr


sts graph, by(colch_tp)  failure ///
 $kmstuff  risktable(, format(%3.0f)) ///
	xtitle("Days") ///
	ytitle("Cumulative Mortality (%)") scheme(s1mono) ///
	title("Time to Death (Propensity Score Analysis)", size(*0.8) ///
	box bexpand bmargin(0 0 0 0)) ///
	text(.7 17 "P value = `spval'")
graph export colch_propensity_score_iptw_death_only.png, replace


*-------------------------------------------------------------------+
* End Survival Analysis (21-day mortality)
*-------------------------------------------------------------------+
```

The authors state that patients with a clinical scenario of COVID infection were included in the analysis even if the first swab was negative, if then the second swab was positive. In which ward were these patients admitted? If they were admitted to a COVID unit it is likely that the result of the second swab was affected by the presence of the virus in the ward.

**We understand the Reviewer’s concern. However, false-negative RT-PCR test from swab tests have been well documented. If initial testing was negative, COVID-19 diagnosis was made based on the presence interstitial pneumonia and then confirmed in the next days with a repeated swab test. Therefore, the possibility that the pneumonia was due to a subsequent SARS-CoV2 infection is very unlikely.**

Please codify all the abbreviation (i.e. SARS-CoV2, SARS and MERS are never stated in full).

**According to the Reviewer’s suggestion, we codified all the abbreviations.**

The authors state that increased serum concentration of cytochines are found in SARS and MERS patients as well. I would suggest to discuss the evidences supporting the use of colchicine in SARS and MERS.

**In the revised discussion, we included a sentence specifying that “likewise colchicine** **would be a useful drug also to treat a possible resurgence of SARS and MERS.”**

In the methods section authors state that patients included in the current analysis were admitted at Parma University Hospital, but a share of patients were outpatients. Please clarify.

**We thank the Reviewer for pointing this out. In the revised manuscript, we clarified that patients where were followed-up (Line 110). In Line 113, we described the study cohort more in detail.**

The treatment of acute pericarditis requires a colchicine dose reduction to 0.5 mg/day in case of body weight < 70 Kg. Was this dose reduction considered?

**We considered a dose reduction for liver or chronic kidney disease. We adopted as reference dose the one used in autoinflammatory disease, like Mediterranean Fever or Behcet disease where the standard dose is 1 mg once daily in adults patients.**

According to table 1, 33 patients had IL-6 dosed at admission but it is not reported the average value.

**The IL-6 values are reported in the results section, Lines 267-269. “Unlike patients in the control group, those who received colchicine had IL-6 serially measured because of the physicians’ expectation that the drug would decrease IL-6 levels”, patients in the control group did not have IL-6 values measured. IL-6 levels in the colchicine group are reported in Figure 4. We corrected the typing error in Table 1.**

Colchicine may increase the risk of bacterial pneumonia. In this view, even though I admit that the diagnosis is very challenging in this scenario, authors should state if any patient had a suspicion of bacterial pneumonia at baseline during the study follow-up.

**This is an important issue. Almost all the patients received antibiotic therapy at the time of COVID-19 diagnosis, but we cannot formally rule out the presence of superimposed bacterial pneumonia. However, in light of the reduced mortality in patients who received colchicine, this hypothesis seems unlikely.**

**We thank the Reviewer for the constructive comments that helped us improving our paper.**
